# The role and its mechanism of intermittent fasting in tumors: friend or foe?

**DOI:** 10.20892/j.issn.2095-3941.2020.0250

**Published:** 2021-02-15

**Authors:** Xu Zhao, Jing Yang, Ruoyu Huang, Mengmeng Guo, Ya Zhou, Lin Xu

**Affiliations:** 1 Special Key Laboratory of Gene Detection and Therapy & Base for Talents in Biotherapy of Guizhou Province, Zunyi 563000, China; 2 Department of Immunology, Zunyi Medical University, Zunyi 563000, China

**Keywords:** Intermittent fasting, energy metabolism, tumor, immune escape, immunotherapy

## Abstract

Intermittent fasting (IF) is becoming a prevailing topic worldwide, as it can cause changes in the body’s energy metabolism processes, improve health, and affect the progression of many diseases, particularly in the circumstance of oncology. Recent research has shown that IF can alter the energy metabolism of tumor cells, thereby inhibiting tumor growth and improving antitumor immune responses. Furthermore, IF can increase cancer sensitivity to chemotherapy and radiotherapy and reduce the side effects of these traditional anticancer treatments. IF is therefore emerging as a promising approach to clinical cancer treatment. However, the balance between long-term benefits of IF compared with the harm from insufficient caloric intake is not well understood. In this article, we review the role of IF in tumorigenesis and tumor therapy, and discuss some scientific problems that remain to be clarified, which might provide some assistance in the application of IF in clinical tumor therapy.

## Introduction

**Figure 1:**
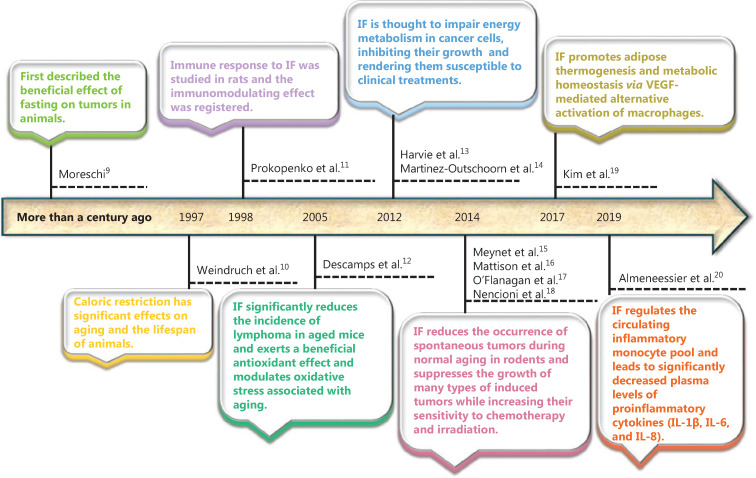
Overview of the major progression of intermittent fasting research.

Intermittent fasting (IF) is a diet-based therapy that alternates between fasting and free feeding/eating for a period of time. This practice was developed by people seeking practical and relatively safe fasting methods to achieve daily caloric restriction. IF regimens generally include short-term intermittent fasting [such as 16–18 h of daily fasting, alternate-day fasting or 5:2 IF (2 days a week)], long-term intermittent fasting, and so on^1–8^ (**Table 1**). More than a century ago, Moreschi^9^ first described the beneficial effects of fasting and restricting caloric intake on tumors in animals. In 1997, Weindruch et al.^10^ found that reducing food availability over a lifetime (caloric restriction, CR) had significant effects on aging and the lifespan of animals (**Figure 1**). Follow-up studies have subsequently showed that IF over a period of 12 h to several weeks can prevent diseases and delay aging in many organisms (such as bacteria, yeasts, worms, and mice). Specifically, IF can improve the function of the entire body, including tissues and organs. Moreover, after conducting an IF regimen, some tissues and organs are more resistant to a variety of harmful stimuli that involve metabolic, oxidative, ionic, traumatic, and proteotoxic stresses^21^. Animal model studies of experimental diseases have further shown that IF can alleviate the development of many chronic diseases, including obesity, diabetes, vascular diseases, cancer, and neurodegenerative diseases^22^.

**Table 1:** The type and definition of nutritional intervention regimens

Intervening regimen	Definition	Reference
Intermittent energy restriction	Restricting energy intake to 60%–75% below energy requirements for short periods, followed by periods with normal energy intake [e.g., the 5:2 diet (consisting of 5 days of libitum feeding and 2 days of a very-low-calorie diet per week)]	^1^
Short-term intermittent fasting	Temporarily fasting (water only), typically for a period between 24 and 48 h	^1^
Fasting-mimicking diet (FMD)	A plant based, calorie-restricted, low sugar, low protein, and high-fat dietary composition administered cyclically and alternated with refeeding periods sufficient to prevent or minimize lean body mass loss	^2^
Calorie restriction (CR)	A dietary strategy usually based on decreasing the calorie intake (about 20%–40% of the *ad libitum* diet) without challenging the intake of essential nutrients	^3^
Ketogenic diet	An ultra-low carbohydrate diet that does not directly restrict calories or require periods of fasting. Successful generation of ketone bodies can suppress appetite and reduce plasma glucose concentrations in cancer-free individuals	^4^
Long-term intermittent fasting	With durations between 5 and 21 days can be successfully repeated in the course of a year	^5^

**Figure 2:**
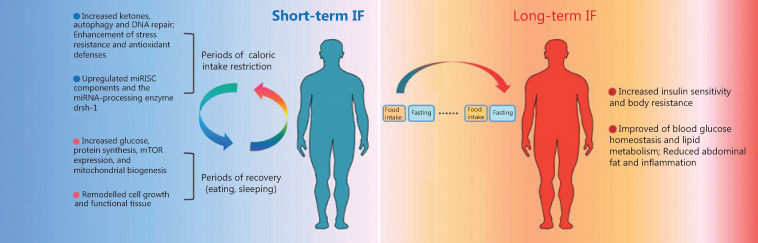
The effect of different periods of intermittent fasting (IF). In the early stage of IF, the effect of IF mainly occurs through ketogenesis, which manifests as increased ketone body production, autophagy, and DNA repair, as well as enhanced anti-stress abilities and antioxidant defense. During periods of recovery (including eating and sleeping), the main energy sources of cells are converted from ketones to glucose, which leads to increased glucose, mTOR expression, and mitochondrial biogenesis, enhanced ability to synthesize intracellular proteins, and remodeled growth and functions. During the long-term adaptation period, the insulin sensitivity of cells and body resistance to stress are increased, blood glucose homeostasis and lipid metabolism are further improved, and abdominal fat and inflammation are reduced.

From a mechanistic perspective, studies have demonstrated that the effect of IF is related to the adaptive energy metabolic response of organs, tissues, and cells triggered by IF (mainly the metabolic conversion from glucose to ketone bodies as an energy source), which manifests as increased ketone body production, autophagy, DNA repair, and anti-stress abilities, and antioxidant defense is enhanced in the early stage of IF. During periods of recovery (including eating and sleeping), the conversion from ketones to glucose as the main energy source of cells results in an enhanced ability to produce glucose and synthesize intracellular proteins and increased mTOR expression and mitochondrial biogenesis. During the long-term adaptation period, the insulin sensitivity of cells and body resistance to insulin are increased, blood glucose homeostasis and lipid metabolism are further improved, and abdominal fat and inflammation are reduced^23–26^ (**Figure 2**). These processes are accompanied by alterations in the insulin-like growth factor-1 (IGF-1)/mTOR signaling pathway, a decrease in leptin levels, an increase in adiponectin levels^27–29^, an improvement in anti-stress ability, a decrease in free radical production, growth and functional remodeling, and an increase in body resistance to stress^30,31^.

A recent study reported that the miRNA machinery, particularly the miRNA-processing enzyme Drosha/drsh-1, is involved in fasting-induced changes in gene expression and IF-induced longevity. This study found that miRISC components (alg-1, alg-2, ain-1, and ain-2) and the miRNA-processing enzyme, drsh-1, are upregulated by fasting, suggesting that the miRNA machinery is activated in response to fasting (**Figure 2**). The expression of miRNA machinery proteins (Argonaute, Dicer, and Drosha) in mouse adipose tissues has been reported to decrease with aging, and these decreases are suppressed by CR. Moreover, miRNA array analyses revealed that the expression levels of numerous miRNAs changed after 2 days of fasting^32^. These results indicate that components of the miRNA machinery, especially the miRNA-processing enzyme, drsh-1, and miRNA molecules, might play important roles in mediating IF-induced longevity *via* the regulation of fasting-induced changes in gene expression.

## Intermittent fasting and tumorigenesis

Recent studies have shown that IF can affect the energy metabolism of tumor cells and inhibit tumor cell growth, and can also improve the function of immune cells and promote antitumor immune responses, which indicates that IF has potential value in tumor immunotherapy^33^.

### IF and tumor growth

**Figure 3:**
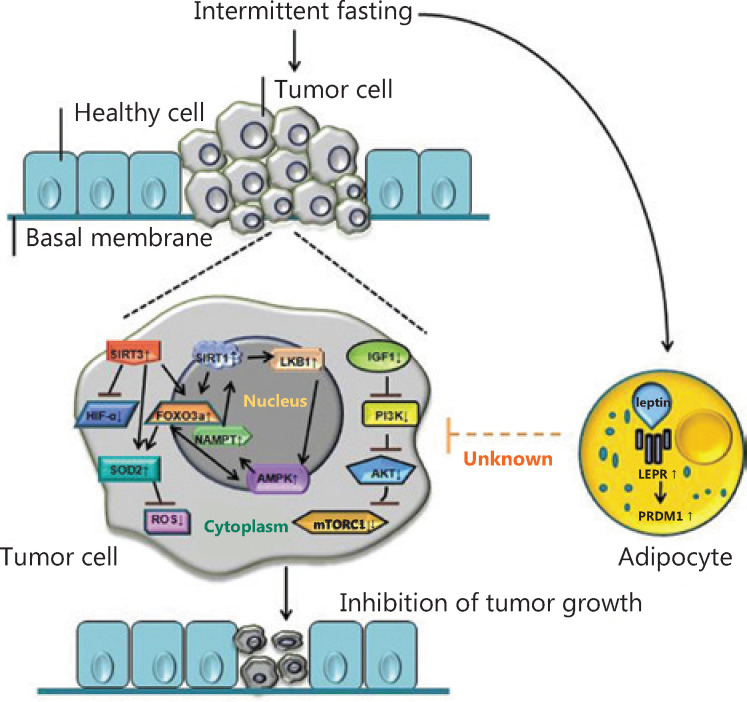
The molecular mechanism by which intermittent fasting (IF) affects tumor cell growth. Mechanistically, IF inhibits the IGF-1/AKT and mTORC1 pathways in tumor cells, while the AMPK, SIRT1, and SIRT3 pathways are activated. In addition, AMPK and SIRTs depend on each other in IF-associated metabolic adaptation. However, AMPK can induce activation of SIRT1 through NAMPT, while SIRT1 can activate AMPK through LKB1 regulation. FOXO3a (a downstream molecule of SIRT1 and SIRT3) and AMPK can each enhance the other’s transcriptional activities. Furthermore, SIRT3 inhibits tumor growth by activating FOXO3a and the expression of superoxide dismutase (SOD), thereby reducing the level of reactive oxygen species and negatively regulating the expression of HIF-1α. SIRT3 activates SOD2 *via* upregulation of FOXO3a. Furthermore, IF can selectively inhibit tumor growth by upregulating LEPR and its downstream signaling pathway protein, PRDM1.

The molecular mechanism by which IF inhibits tumor cell growth is as follows. CR induced by IF inhibits the IGF-1/AKT and mTORC1 pathways in tumor cells, while adenosine 5´-monophosphate (AMP)-activated protein kinase (AMPK), which is dependent on the nicotinamide adenine dinucleotide coenzyme deacetylase-1 (Sirtuin-1, SIRT1) and SIRT3 pathways, is activated, thus hindering tumor cell growth. Notably, AMPK and SIRTs depend on each other in IF-associated metabolic adaptation^34,35^. AMPK can induce activation of SIRT1 through nicotinamide phosphoribosyltransferase (NAMPT), while SIRT1 can activate AMPK through liver kinase B1 (LKB1) regulation. In addition, FOXO3a (a downstream molecule of SIRT1 and SIRT3) and AMPK can each enhance the other’s transcriptional activities^36^. SIRT3 inhibits tumor growth by activating FOXO3a and the expression of superoxide dismutase 2 (SOD2), thereby reducing the level of reactive oxygen species (ROS)^37-39^ and negatively regulating the expression of hypoxia inducible factor-1α (HIF-1α)^40,41^. SIRT3 activates SOD2 *via* upregulation of FOXO3a^42-45^. Furthermore, IF can selectively inhibit tumor growth by upregulating leptin receptor (LEPR) and its downstream signaling pathway protein, PR/SET domain gene family 1 (PRDM1)^46^ (**Figure 3**).

However, the major decreases in glucose, insulin, and IGF-1 caused by fasting, which is accompanied by cell death and/or atrophy in a wide range of tissues and organs, including the liver and kidneys, is followed by a period of abnormally high cellular proliferation in these tissues, driven in part by the replenishment of growth factors during refeeding. When combined with carcinogens during refeeding, this increased proliferative activity can increase carcinogenesis and/or precancerous lesions in tissues, including the liver and colon^47^. In addition, some previous studies have shown that IF fails to inhibit the formation of carcinomas in mice^48-50^. Together with these studies, we therefore hypothesize that excessive use of IF may have the opposite effect in inhibiting the occurrence of tumors. To summarize, it is suggested that the mechanism by which IF inhibits tumor growth is complex, and the specific molecular mechanisms that contribute to specific tumors may be different when different IF or CR regimens are implemented.

### IF and tumor immunity

Immune escape is a key factor in tumorigenesis. Studies have shown that IF can affect the development and function of a variety of immune cells, thereby regulating antitumor immune responses and affecting tumorigenesis.

**Figure 4:**
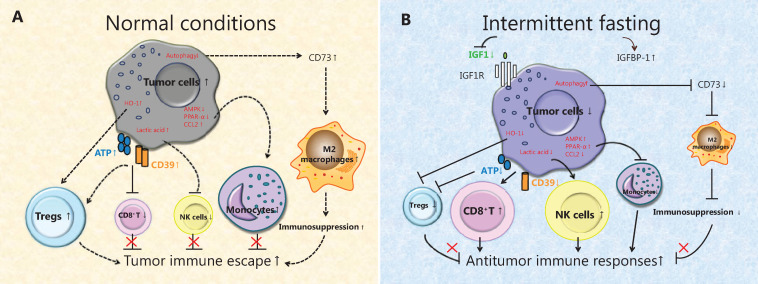
The effect of intermittent fasting (IF) on tumor immune responses. (A) Under normal conditions, the low autophagy level of tumor cells promotes the expression of CD73 and adenosine, which in turn affects the M2 polarization of tumor-associated macrophages (TAMs). Moreover, the CD39 expression and extracellular ATP increase, which stimulate Tregs and inhibit the functions of cytotoxic T lymphocytes (CTLs). In addition, heme oxygenase 1 is highly expressed in tumors and can inhibit apoptosis and immunostimulatory effects. The lactic acid produced by tumor glycolysis inhibits the function of natural killer (NK) cells. (B) During IF, programmed cell death of tumor cells increases *via* autophagy and then reduces the expression of CD73 and adenosine in the tumor microenvironment, which inhibits the M2 polarization of TAMs. Furthermore, the expression of CD39 in tumor cells and the accumulation of extracellular ATP are inhibited, thus inhibiting the function of regulatory T cells and stimulating the function of CTLs. Moreover, IF can reduce the lactic acid level, thereby restoring the function of NK cells. Activation of the energy effectors AMPK and PPAR-α (a downstream molecule of AMPK) inhibit the production of CCL2, thereby reducing the migration of monocytes from the bone marrow into the tumor microenvironment.

IF affects antitumor immunity mainly by increasing the self-renewal ability of hematopoietic stem cells and improving immunosuppression. Moreover, the conversion of energy metabolism results in dramatic downregulation of IGF-1 and upregulation of insulin-like growth factor binding protein-1^51^ and increased tumor cell autophagy and programmed cell death. These effects are accompanied by a decrease in extracellular nucleoside triphosphate diphosphohydrolase expression and ATP accumulation, thus inhibiting regulatory T cells (Tregs) and stimulating cytotoxic T lymphocyte (CTL) functions, which enhances antitumor immune responses^52^. Moreover, high expression of heme oxygenase-1 (HO-1) in tumors can inhibit tumor cell apoptosis and immunostimulatory effects^53^. Notably, IF can decrease the expression of HO-1 in tumor cells and increase tumor cell apoptosis, as well as enhance stimulation of CD8^+^ T cells by reducing the caloric supply, which forms a positive feedback loop of IF-induced CD8^+^ T cell-medicated killing of tumor cells (**Figure 4**)^54^.

Furthermore, IF can also regulate antitumor immune responses by affecting the function and polarization of innate immune cells, such as natural killer (NK) cells and tumor-associated macrophages (TAMs)^55^. Lactic acid derived from tumor cells can directly inhibit NK cell-mediated killing and also increase the number of myeloid-derived suppressor cells (MDSCs) to indirectly inhibit NK cell functions. IF can reduce the production of lactic acid by tumor glycolysis, thereby restoring the function of NK cells, reducing the enrichment of MDSCs, and inhibiting tumor growth^56^. In addition, the occurrence of tumors is also closely related to polarization of local TAMs. Recent studies have shown that IF can reduce the metabolism and inflammatory activity of monocytes due to the activation of peroxisome proliferator-activated receptor-α (PPAR-α) during fasting, and also enhance repair and immune surveillance in the body *via* activation of AMPK. Furthermore, IF inhibits the production of chemokine C-C motif ligand 2 (CCL2) to reduce the number of inflammation-related monocytes in the blood and tissues^57^. Moreover, IF can also inactivate the JAK1/STAT3 signaling pathway and reduce the expression of CD73 and adenosine in the tumor microenvironment, which in turn affects the M2 polarization of TAMs and inhibits tumor growth^58^ (**Figure 4**).

The above studies suggest that IF can cause favorable changes in the immune cell population, but there are results that contradict these studies. For example, IF in a lupus mouse model leads to the expansion of Tregs^59^. Moreover, IF can exacerbate acute immunity and behavioral sickness in response to stimulation with the viral mimic, poly(I:C)^60^. These findings suggest that the effect of a specific IF regimen on the development and function of immune cells is complex and remains to be elucidated.

## IF and tumor therapy

### IF, chemotherapy, and radiotherapy

Previous studies have shown that fasting cycles combined with chemotherapy (CT) are viable and might slow tumor progression and reduce CT-induced side effects in some patients with different types of cancer. The beneficial therapeutic effect of IF combined with CT on tumors indicate that IF enhances the therapeutic effect of CT on tumors^61,62^ and also significantly ameliorates the inflammation caused by CT^63-69^. Early studies suggested that IF can produce effective immunity and reduce the incidence of infection and febrile neutropenia. Moreover, in animal models of systemic bacterial infection, IF can inhibit inflammation and protect cognitive function^70,71^. Further analysis has shown that IF can inhibit the expression of proinflammatory cytokines, including RANTES/CCL5, TNF-α, IL-1β, IL-6, and IL-18, and the inflammatory body proteins NLRP1 and NLRP3 by activating NF-κB, thereby reducing the incidence of inflammation^72,73^.

However, the exact mechanism involving the effects of IF combined with CT might also be complex due to the varied influence of IF on gene expression. IF before CT has been shown to protect the host from treatment toxicity by reducing the expression of some oncogenes, such as RAS and the AKT signaling pathway. This reduction is mediated by decreases in circulating IGF-1 and glucose. Moreover, IF-induced CR activates other oncogenes in cancer cells, induces autophagy, and decreases cellular growth rates while increasing the sensitivity of tumor cells to antimitotic drugs. In addition, the effect of IF combined with CT on tumor growth may depend entirely on the cellular immune system, because CT combined with IF cannot control tumor growth in nonthymic mice due to the lack of T lymphocytes^74^.

Similarly, some studies have shown that IF increases cancer sensitization to radiotherapy (RT) and extends the survival of starved experimental animals after different IF regimens. For example, IF improves the survival of mice with orthotopic pancreatic tumors subjected to lethal abdominal radiation compared with controls with free access to food. Moreover, IF does not affect radiation therapy-mediated tumor cell killing and enhances γ-H2AX staining after radiation therapy, suggesting an additional mild radiosensitizing effect^75^. Importantly, preclinical and some preliminary clinical data support the hypothesis that IF could be utilized as a complementary treatment to improve the outcome after RT, both in terms of improved tumor control and a reduced probability of normal tissue complications^76,77^.

Although IF combined with these traditional anticancer therapies has shown promising results in these basic studies, more preclinical experimental data are still needed because clinical relevance remains low due to insufficient human data, including few clinical outcome studies, a lack of safety data, and almost no randomized controlled trials^78^. Future studies therefore need to focus on safety, and ultimately increase benefits to current therapies associated with IF. Moreover, the potential of IF to enhance the response to lower doses of CT and radiation therapy also should be further investigated.

### IF and tumor immunotherapy

Presently, there are no reports about IF and tumor immunotherapy. In the tumor microenvironment, IF can reduce glucose uptake and glycolysis in tumor cells. However, effector T cells of antitumor immune responses also rely on glycolysis to maintain their clonal expansion and function. Importantly, studies have shown that immune checkpoint blockade (ICB) therapy with PD-L1 antibodies selectively protects T cells from decreased glucose utilization in the tumor microenvironment^79^, which suggests that ICB combined with IF may become a promising clinical tumor treatment strategy. However, IF can reduce the level of circulating glucose and slow the growth of tumor cells^80^. ICB is used to improve the energy metabolism of T cells and maintain their function^81^ to achieve an improved effect of clinical tumor immunotherapy. However, considering the complexity of the energy metabolism mechanism triggered by IF and the differences in ICB immunotherapy methods, more prospective studies are needed to characterize the regimens and effects of IF combined with tumor immunotherapy.

### IF and other therapies

One recent study showed that the combination of IF and vitamin C was a promising intervention with low toxicity, which could be tested in randomized clinical trials against colorectal cancer and possibly other tumors with KRAS mutations. Specifically, the anticancer activity of vitamin C is limited by the upregulation of HO-1. However, IF selectivity reverses vitamin C-induced upregulation of HO-1 and ferritin in KRAS-mutant cancer cells, consequently increasing reactive iron, oxygen species, and cell death, an effect that is further potentiated by CT^82^. These findings indicate that IF combined with noncytotoxic compounds could be novel and promising treatments of tumors. In addition, the related progression on IF combined with tumor therapy was listed in the **Table 2**.

**Table 2:** Overview of intermittent fasting (IF) combined with tumor therapy

Year	Disease(s)	Dietary regimen	Outcome	Mouse strains	Reference
2020	Colorectal cancer and other KRAS-mutant tumors	Standard feeding or on the last day of the first IF cycle, vitamin C (4 g/kg in saline) began *via* intraperitoneal injection twice a day, with at least 6–8 between the 2 daily administrations	The combination of IF and vitamin C represented a promising low toxicity intervention	NOD scid, BALB/c mice	^82^
2019	Orthotopic pancreatic tumors	Fed or fasted for 24 h and then exposed to total abdominal radiation at a dose of 11.5 Gy	IF improved the survival of mice with orthotopic pancreatic tumors subjected to lethal abdominal radiation	C57BL/6J mice	^83^
2017	Colon cancer	24 h fasting on alternate days for 2 weeks	IF inhibited colon cancer growth and decreased the production of extracellular adenosine by cancer cells by suppressing CD73 expression	BALB/c mice	^84^
2016	Fibrosarcoma	48 h fasting (water only) once *vs*. *ad libitum* feeding; animals were injected with mitoxantrone or oxaliplatin at the end of fasting	IF improved the efficacy of chemotherapy in an immune system-dependent and autophagy-dependent fashion	C57Bl/6 and athymic nude mice	^85^
2016	Breast cancer; melanoma	48–60 h fasting (water only) or a 96 h FMD once a week for 2–4 weeks *vs*. *ad libitum* feeding; animals were injected with chemotherapy at the end of each fasting and/or FMD cycle	IF slowed tumor progression when combined with doxorubicin or cyclophosphamide, expanded lymphoid progenitors and boosted anticancer immunity	BALB/c, C57Bl/6 and athymic nude mice	^86^
2015	Colon cancer	48 h fasting (water only) once a week for 2 weeks, 24 h prior to and 24 h after oxaliplatin injection *vs*. *ad libitum* feeding	IF improved the anticancer effects of oxaliplatin, exerted anti-Warburg effects and promoted oxidative stress and apoptosis in cancer cells	BALB/c mice	^87^
2015	Lung cancer; colorectal cancer	48 h fasting (water only) once a week for 3 weeks with daily treatment with crizotinib or regorafenib *vs*. *ad libitum* feeding	IF improved the clinical activities of crizotinib and regorafenib	Athymic nude mice	^88^
2012	Mesothelioma; lung cancer	48 h fasting (water only) given once a week for 3 weeks, 32 h prior to and 16 h after cisplatin injection *vs*. *ad libitum* feeding	IF sensitized human mesothelioma and lung cancer xenografts to cisplatin	Nude mice	^89^

## Conclusions

**Figure 5:**
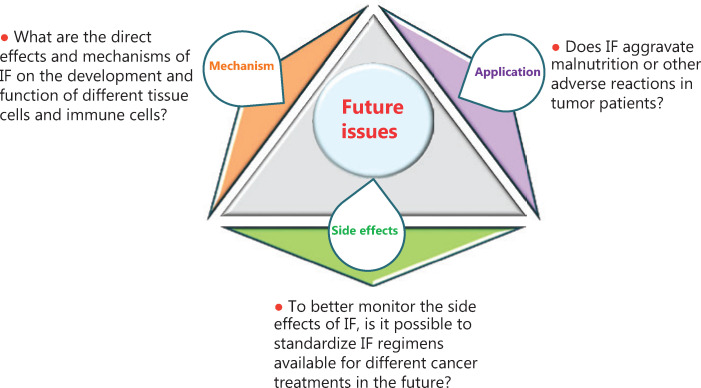
A diagram of future intermittent fasting issues.

Current studies have shown that IF possesses a wide range of effects that improve energy metabolism and the occurrence of many diseases; IF has a crucial effect on tumor immune responses, suggesting that IF is emerging as a promising strategy in clinical tumor therapy. However, because of the complexity of the effects of IF on tissues and cell energy metabolism, there are still many scientific challenges to be further addressed. In the present review, we summarized three aspects of these scientific problems (**Figure 5**). (1) Regarding the mechanism, what are the direct effects and mechanisms of IF on the development and function of different tissue-specific cells and immune cells? For example, one recent study showed that 30-day IF was associated with an anticancer serum proteomic signature, upregulated key regulatory proteins associated with glucose and lipid metabolism, and affected the circadian clock, DNA repair, cytoskeletal remodeling, the immune system, and cognitive function, and resulted in a serum proteome that was protective against cancer, metabolic syndrome, and inflammation. Furthermore, it is important that there are some different nutritional interventions (**Table 1**), which may lead to different effects on physiological indices including blood glucose, triglycerides, growth hormone, insulin, and insulin-like growth factor 1 (IGF-I). Different clinical treatment strategies also might affect the ultimate effects of IF in cancer patients. Further research is therefore needed before the use of IF as an intervention can be recommended to increase the quality of life for tumor patients. (2) Regarding side effects, the different influences of distinct IF regimens on the energy metabolism of tumor patients remain largely unknown. To better monitor the side effects of IF, is it possible to standardize IF regimens for different cancer treatments? One study reported that it will be necessary to test and standardize IF regimens by methodologies that are similar to those performed for the approval of drugs by the FDA, to allow these interventions to be implemented by a large portion of the population. (3) Regarding application, does IF exacerbate malnutrition or other adverse reactions in tumor patients? Some studies report that malnutrition and sarcopenia frequently occur in cancer patients and have a negative effect on clinical outcomes. The reason may be driven by inadequate food intake, decreased physical activity, and catabolic metabolic derangements. The process regarding the application of IF in cancer patients should therefore be more rigorous, and examination of major adverse clinical events (including malnourishment, cachexia, possibly a weakened immune system, and increased susceptibility to certain infections, which need to be carefully monitored) are a vital step in determining whether nutritional intervention is actually beneficial. In addition, because existing research has shown limited safety outcomes, the findings are only useful in developing longer-term trials. Therefore, future studies will have to take into consideration the risk of malnutrition and sarcopenia, and the immunological and metabolic state of the enrolled patients.

In summary, IF has emerged as a promising and powerful tool in clinical tumor therapy. However, IF is a double-edged sword that can affect tumorigenesis, increase immune responses, and alter the energy metabolism of tumor patients. Additionally, there is a notable lack of research on the effects of IF combined with tumor immunotherapy and gene therapy. Thus, further research is needed before the use of IF as an intervention can be recommended to increase the quality of life for tumor patients. We believe that with further elucidation of the mechanism of IF accompanied by the development of molecular biology, systems biology, and mega data, specifically regarding the relationship between IF and tumor immune responses, IF will result in new strategies for clinical tumor immune prevention and treatment in the near future, with wide application prospects.
